# A Framework for Analyzing Fraud Risk Warning and Interference Effects by Fusing Multivariate Heterogeneous Data: A Bayesian Belief Network

**DOI:** 10.3390/e25060892

**Published:** 2023-06-02

**Authors:** Mianning Hu, Xin Li, Mingfeng Li, Rongchen Zhu, Binzhou Si

**Affiliations:** School of Information and Network Security, People’s Public Security University of China, Beijing 100038, China; 201821450006@stu.ppsuc.edu.cn (M.H.);

**Keywords:** telecom fraud, Bayesian network, early warning framework, multiple heterogeneous data

## Abstract

In the construction of a telecom-fraud risk warning and intervention-effect prediction model, how to apply multivariate heterogeneous data to the front-end prevention and management of telecommunication network fraud has become one of the focuses of this research. The Bayesian network-based fraud risk warning and intervention model was designed by taking into account existing data accumulation, the related literature, and expert knowledge. The initial structure of the model was improved by utilizing City S as an application example, and a telecom-fraud analysis and warning framework was proposed by incorporating telecom-fraud mapping. After the evaluation in this paper, the model shows that age has a maximum sensitivity of 13.5% to telecom-fraud losses; anti-fraud propaganda can reduce the probability of losses above 300,000 yuan by 2%; and the overall telecom-fraud losses show that more occur in the summer and less occur in the autumn, and that the Double 11 period and other special time points are prominent. The model in this paper has good application value in the real-world field, and the analysis of the early warning framework can provide decision support for the police and the community to identify the groups, locations, and spatial and temporal environments prone to fraud, to combat propaganda and provide a timely warning to stop losses.

## 1. Introduction

With the rapid development of the information era, telecom fraud has expanded from the traditional communication industry to other channels that deliver fraudulent information through Over The Top (OTT) platforms such as QQ, WeChat, and microblogs, and use them as a medium to spread fraudulent information, which seriously damages people’s lives and property safety [1]. In 2021 alone, a total of 394,000 cases of telecommunication network fraud were solved and 634,000 suspects were arrested nationwide, which represents a year-on-year increase of 28.5% and 76.6% [2]. Therefore, prompt action is needed to tackle the issue of telecom-fraud governance.

In recent years, China has diversified its approach to governing telecom fraud. Among policy developments, in 2015, the Chinese Ministry of Industry and Information Technology proposed the “Management of Communication Short Message Service” document to strengthen governance of SMS, while implementing a real-name system for cell phones to address anonymous communication in the traditional communication industry. The Anti-Telecommunication Fraud Law, introduced in 2022, formally incorporated the telecom-fraud industry chain into criminal crackdown governance, marking a milestone [3]. In early academic research, Jiang [4] et al. used communication patterns between fraudsters and victims learned from SMS data to train a recognition model that fused fraudster features, achieving good results in the defense against communication fraud. Yang [5] et al. used a hierarchical subspace approach to improve the weights of decision trees using different tree classification capabilities, with the accuracy of the improved random forest algorithm reaching nearly 90%. In new research on telecom fraud, Hu [6] et al. proposed a technical means to integrate multiple strategies to improve model identification performance, taking fraudulent short messages on OTT platforms as the governance object. Jiang [7] et al. used a social network analysis method to study organizational structure, excavating the core members and important intermediaries of criminal groups for crackdowns on telecom-fraud groups. Ni [8] et al. built a Bayesian network model due to the causal analysis capability of Bayesian networks, selected the characteristic perspective of fraud victims, learned the model parameters (including posterior distribution values) from telecom-fraud-related data, and finally proved the applicability of the model based on causal inference capability, such as situational inference and sensitivity verification. The current problems in telecom-fraud technology research and application include: (1) not answering cause-and-effect questions such as “what, if?”; (2) there exists no quantitative research on external dynamic influences, such as major public health emergencies, police crackdown campaigns, and important time points; (3) failure to quantitatively assess the risk value and realize the risk warning for potential victims; and (4) the research mostly takes limited data (fraudulent SMS, suspicious phone numbers, etc.) as the object to provide governance ideas, and fails to break the barriers between multiple data.

Therefore, this paper selects 14 types of features from multiple heterogeneous data and combines them to achieve a quantitative representation of telecom-fraud features by combining them. Specifically, this paper designs and builds a Bayesian network-based telecom-fraud consequence model to capture and quantify the individualized background features of telecom fraud (e.g., education level, financial industry coverage, and regional functional type), the process of telecom fraud (fraud type, contact mode), the consequences of telecom fraud (loss situation, recovery period), and the causal relationships between the factors.

## 2. Methods

### 2.1. Choose a Bayesian Network

Currently, telecom fraud is the result of complex and nonlinear interactions among multiple factors throughout the entire process [9]. Directly extracting data from multivariate heterogeneous data sources may increase the uncertainty of the training model as the original data may contain noise [10]. Integrating expert knowledge with machine learning models to interpret the data is an effective attempt. However, most machine learning models suffer from a lack of “interpretability”, e.g., the models remain black boxes, unable to explain the reasons behind predictions and decisions, thus losing user trust and impeding the process of identifying and addressing issues [11]. Therefore, the use of causal modeling can be a good solution to these problems. However, computer systems currently need to answer, “what if?”, interventional, retrospective, or explanatory questions when solving cause-effect problems.

At present, Pearl [11] divides the causal hierarchy into three levels, and different levels can answer different types of causal information questions. As shown in Table 1, the three levels are defined as Association, Intervention, and Counterfactuals, from the bottom to the top. The first level, Association, is mainly the observation stage, aimed at achieving the discovery of explicit statistical associations among existing data. In more general terms, it can be called statistical analysis. The second level deals with Intervention—the act of setting variables to specific values. Thus experimental results make it possible to discover the causal relationships between the considered variables. The third level deals with Counterfactuals, and it focuses on the hypothetical change of variables in the past in order to engage in retroactive reasoning, considering what could have happened [12,13]. As the top level, the Counterfactuals stage involves solving both conventional questions and association questions. In his paper, Pearl [11] explains why many correlation-based machine learning models are unable to reason about actions, experiments, and causal explanations based on a three-level causal hierarchy, thus illustrating the powerful causal analysis capabilities of Bayesian networks and the preference for causal modeling when faced with complex problems. The feasible approaches among the three technical approaches in answering different types of cause-effect questions are also shown in Table 1.

### 2.2. Introduction to Bayesian Networks

A Bayesian network, also known as a belief network, is a probabilistic graphical model that represents a random variable and its conditional distribution through a directed acyclic graph. The Bayesian network is composed of two components: qualitative and quantitative. The qualitative part is a directed acyclic graph, where nodes represent random variables and arcs represent dependencies or causal relationships between variables. There are two types of nodes in the Bayesian network: leaf nodes, which have only parent nodes and no child nodes, and root nodes, which have only child nodes and no parent nodes. The quantitative part refers to the prior probability of each parent node and the posterior probability of each child node in the statistical inference network based on the constructed Bayesian network nodes and directed links. For example, there are n random variables $V_{1},V_{2},\cdots ,V_{n}\;$ and an acyclic graph with n numbered nodes, and it is assumed that node $j\;\left(1<j<n\right)$ in the acyclic graph is related to the variable $V_{j}$. Then, the joint probability distribution of this set of random variables is given by:

(1) $$P\left(V_{1},V_{2},V_{3},\cdots ,V_{n}\right)=\prod_{j=1}^{n}P\left(V_{j}|parent\left(V_{j}\right)\right)$$

Among them, $parent\left(V_{j}\right)$ represents the set of all variables that originate arrows (oriented edges) ending in node-variable $V_{j}$. 

### 2.3. Multivariate Heterogeneous Data-Fusion Method

**Figure 1:**
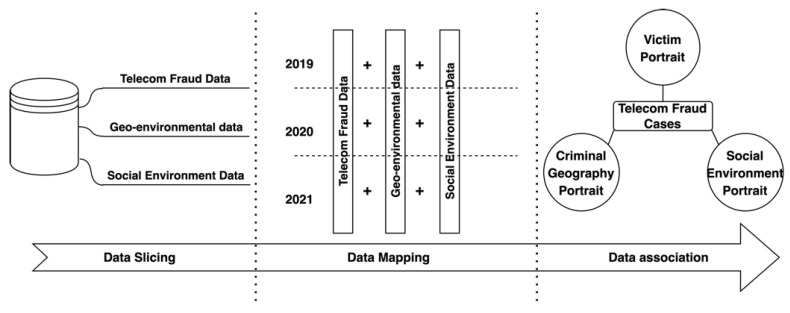
Flowchart of Multiple Heterogeneous Data-Fusion Method for Telecom-Fraud Alerting.

Data-fusion technology was first developed gradually in the United States in the military field in order to combine multiple sensory data to meet the needs of military operations. For this purpose, the U.S. also established a special expert group on data-fusion technology in 1984. Later, with the intervention of knowledge-based reasoning and other approaches, this technology was made more general and applied in more fields [14]. Data-fusion methods include both traditional methods of weighted average probability statistics and emerging techniques such as convolutional neural networks, D-S evidence inference, and fuzzy computing [15]. In order to improve the accuracy of telecom-fraud analysis and early warning, this paper proposes a multiple heterogeneous data-fusion method based on multiple spatio-temporal dimensions, which slices, maps, and correlates telecom-fraud information, geographic environment information, and social environment information from spatio-temporal dimensions to realize the fusion of multiple heterogeneous data into a multidimensional portrait associated with telecom-fraud cases. As shown in Figure 1, the data-fusion method realizes the full portrait association of telecom-fraud cases.

The use of telecom-fraud data can extract multidimensional victim characteristics and provide victim-level feature information for telecom-fraud consequence models [8]. For crime rasterized regions, the geographic portrait of crime is extracted based on the interaction between space and crime, which can further integrate urban spatial crime characteristics [16]; when using weather conditions and police rate as static factors and police crackdown propaganda and major public health emergencies as dynamic factors, the social environment portrait can be portrayed and the analysis and early warning framework can be improved from both dynamic and static aspects, respectively [17]. In summary, multidimensional data fusion through multidimensional heterogeneous data processing as well as the use of personal portraits, criminal geographic portraits, and social environment portraits, can help analyze and warn against telecom fraud from the whole-process perspective.

## 3. BBN Network Model Design and Construction

### 3.1. Determination of Nodes in the Whole Process of Telecom Fraud

In order to complete the node setting of the Bayesian network, this section combines the whole-process analysis of telecom fraud with the theoretical basis of multivariate heterogeneous data fusion for design. The node design includes five aspects: victim portrait, crime geography environment portrait, social environment portrait, fraud process, and fraud result. Among them, the node status of the BBN-based fraud risk model is constructed through a literature review and expert knowledge, as shown in Table 2.

#### 3.1.1. Victim Portrait Node Status

Crime portraits are widely used in the search for criminal suspects as a traditional technical tool for crime fighting and prevention. With the advent of the information age, portrait technology has also been introduced into the Internet industry and has achieved good results in the work of accurate product pushing and advertising [39]. In constructing the victim portrait, this paper considers various factors. Firstly, gender and age, which are important basic characteristics of people, can influence the polymorphism of criminals’ choice of victims [18]. Women are more emotional in the face of crime compared to men, and the emotional suppression of rationality is more likely to lead to victimization outcomes [19]. Similarly, the age gap, which causes a gap in individual security awareness, can have a significant impact on fraudulent behavior at the subjective level [20]. In addition, the victim’s knowledge level can reflect the level of social awareness at an objective level, and the success rate of being defrauded is significantly lower when the education level is at the bachelor’s degree level and above [21]. Relevant studies have shown that the economic level of a city can lead to changes in the crime rate of a city, so based on the large proportion of foreign population in the research subject City S, this paper defines the economic level of a city by attributing the annual GDP per capita level of the foreign population to the city [23,24].

#### 3.1.2. Criminal Geography Portrait and Social Environment Portrait Node Status

Since Montesquieu pioneered the combination of geography and crime in the mid-18th century, the development of research in criminal geography has spanned nearly 270 years [40]. Among other things, after the introduction of positivism in the 1960s, the research paradigm of criminal geography transitioned from qualitative to quantitative research. When related scholars quantified urban functions in research in criminal geography, they found a clear linear relationship between their distribution and criminal activities [41,42]. Among them, point of interest (POI), as non-geographic information on the map with significant meaning, includes four elements: name, category, coordinates, and classification, which can enrich important environmental information in the region. Therefore, the process when selecting the city functional characteristics to study is: (1) defining a regional functional POI comparison table based on an Internet map vendor’s POI classification criteria (as in Table 3); (2) dividing the city into a cellular hexagon with a side length of 464.1 m and calculating the kernel density value of POIs according to Equations (2)–(6); and (3) calculating the functional type according to quantitative identification method for functional areas proposed by Wang et al. [25,26,27].

(2) $$g\left(a\right)=\sum_{i=1}^{n}\frac{1}{h^{2}}\phi \left(\frac{a-p_{i}}{h}\right)$$

where $g\left(a\right)$ refers to the kernel density estimation function at the location of POI point *a*; *h* is the bandwidth value in the kernel density function also known as the path fading threshold; $p_{i}$ is the location coordinate of the ith POI point within the bandwidth range at *a*; *n* is the total number of POI points within the bandwidth range *h* of POI point *a*; and the ϕ function is chosen as the spatial weight function, corresponding to the equation [25,26,27,43]:

(3) $$\phi \left(\frac{a-p_{i}}{h}\right)=\frac{3}{4}\left[1-\frac{{\left(a-p_{i}\right)}^{2}}{h^{2}}\right]$$

Taking into account the uneven distribution of POI in different regions, hereby the bandwidth *h* is calculated as follows:

(4) $$h=0.9\times \min\left(SD,\sqrt{1/ln^{2}}\times D_{m}\right)\times N^{2}$$

(5) $$SD=\sqrt{\sum_{i=1}^{N}{\left(Distance_{i}\right)}^{2}/N}$$

(6) $$Distance_{i}=2\times R\times \sin^{-1}\left(\sqrt{\sin^{2}\left(\frac{Y_{i}-\bar{Y}}{2}\right)+\cos\left(Y_{i}\right)\times \cos\left(Y\right)\;\sin^{2}\left(\frac{X_{i}-\bar{X}}{2}\right)}\right)$$

where point $\left(\bar{X},\bar{Y}\right)$ denotes the mean centroid coordinates of all POI points; $Distance_{i}\;$ is the distance between the coordinates of the ith POI point and the mean centroid coordinates calculated by Haversine’s formula, and R is the radius of the earth; SD is the standard distance from the mean centroid to all POI points; $D_{m}$ is the median distance between the mean centroid and all POI points.

In addition, expert knowledge shows the importance of the distance of dissuasive institutions and the number of financial institutions in the anti-fraud work. When the distance to the dissuasion institution (police station, street office, anti-fraud center) is closer, the anti-fraud dissuasion is more efficient and the stop-loss effect is better. The more the number of financial institutions, the higher the degree of financial business facilitation in the region, which helps to improve the success rate of elderly victims being cheated [30]. When considering static social environmental factors, two types of environmental factors– diurnal and weather—are selected in this section. This is because poor weather conditions can limit going out behavior and increase online behavior, while dark environments can promote the growth of sensuality and impulsivity and increase the likelihood of being cheated [32,33].

#### 3.1.3. Fraud Process Node Status

Different types of telecom fraud have significant characteristic differences, so telecom fraud is divided into the six types displayed in Table 2. In addition, too many occurrences of repeated telecom fraud will cause victims to fall into panic and cause higher losses, so in this paper, the number of repeated victimization is divided into three types according to the actual situation: 0; 1 to 2; and 3 or more [34]. The choice of whether to report the fraud to the police also directly affects the loss [35]. However, this section does not cover the methods used by fraudsters to make contact with potential victims due to the wide range of contact method routes, with WeChat and cell phones allowing for almost all types of fraud, making it difficult to make effective distinctions.

#### 3.1.4. Fraudulent Results Node Status

The loss case nodes were divided into five classes, as shown in Table 2. Because victims’ mistrust of society lasts for a period of time after being cheated, and the length of time can lead to depression or social unrest (e.g., mass rallies and demonstrations), the analysis of victims’ recovery period was divided into five levels of recovery cycles based on expert experience and literature combing [35,36,37,38].

### 3.2. BBN Network Construction

In this paper, three years of fraud data containing 31,347 cases were collected and screened from the Anti-Fraud Center of City S. To facilitate data retrieval, they have been stored in a relational database management system. Although the majority of the data is complete, there are still some datasets with large amounts of missing data, which may result in errors in the parameters learned by the Bayesian network that need to be cleaned up manually. In addition, the K2 algorithm, which is a classical algorithm that can evaluate the merits of the model structure, can objectively evaluate the Bayesian network structure and use the greedy algorithm to ensure that no information is lost. So, the Delphi method and the K2 algorithm are used in the network construction process to complete the Bayesian network learning.

**Figure 2:**
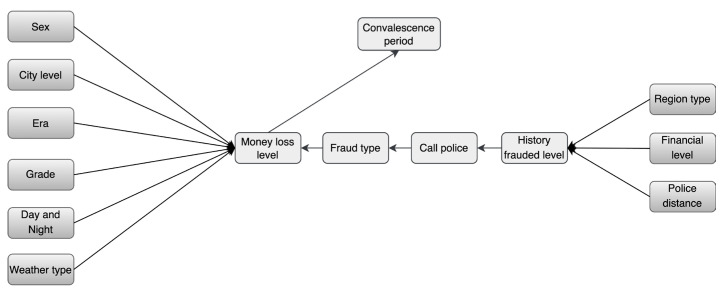
Causality Diagram of Telecom Fraud.

A total of five experts (three academics and two anti-fraud police officers) were invited to participate in the process in order to leverage expert experience. Two of the academics have more than five years of experience in police combating, telecom fraud, and risk assessment, and one academic has a long history of research and in combating governance of geo-information crime. These two officers have seven years of field experience on the front lines of combating telecom fraud. The process is as follows: (1) Initially, the three scholars identified appropriate criteria for assessing telecom fraud, as presented in Table 2 through a review of the existing literature. (2) Then, two grassroots anti-fraud police officers were invited to verify the validity of the parent and child nodes. (3) Finally, a telecom-fraud causality map was drawn based on the experience of the five experts. As shown in Figure 2, in total, there are 13 marginally independent variables and 5 dependent child nodes; 13 oriented edges represent direct causal relationships. In this paper, we used GeNIe software to build the BBN model structure based on the drawn causality diagram, during which the final node names deviated from those in Table 1 due to the limitation of GeNIe software naming formats [44].

### 3.3. Bayesian Network Probability Generation

The prior and posterior probabilities of the variable nodes need to be determined after completing the variable causality graph. The conditional probability table (CPT) of the Bayesian network is derived by learning the prior probability distribution of the variable nodes through expert knowledge and a MAP maximum likelihood estimation algorithm. The general steps are: (1) The historical data of the nodes are processed for machine learning. (2) Experts are asked to modify some probabilities that do not conform to the distribution to complete the maximum practical value of the Bayesian network. (3) In the process of improving the CPT value, three scholars were required to first revise the CPT value of the sub-node based on a literature review and professional knowledge, and then two anti-fraud police officers were asked to further revise the CPT values based on real-world experience. (4) In the process of overcoming the differences in the results, the CPT values were continuously changed until all five experts reached a consensus, and the final model was obtained with 10,488 conditional probabilities. The meaning of the CPT values is explained in the form of examples in Table 4. For example, the child node “loss situation” depends on the parent node “age, city economic level, knowledge level, day and night, weather, fraud type”. Then, the CPT values of loss cases for the parents of post-2000s, low urban economic level, low knowledge level, darkness, good weather, and identity impersonation fraud are (23, 51, 18, 1, 1, 6), which indicate that the conditional probabilities of loss cases correspond to five loss amounts ranging between 23.0%, 51.0%, 18.0%, 1.0%, 1.0%, and 6.0%. The CPTs of other sub-nodes were calculated similarly.

### 3.4. Model Validation

To validate the telecom-fraud consequence model, a total of four experiments were conducted in this section based on the evidence. These include two qualitative analysis validations with extreme conditions testing and scenario reasoning, and two theoretical analysis experiments including sensitivity analysis and partial validation.

#### 3.4.1. Extreme Scenario Testing

In this section, two extreme conditions of the parent nodes are selected to test the response of the Bayesian network. As shown in Table 5, Sex, Era, Grade, City level, Region type, Police distance, Financial level, Day and Night, and Weather type are parents, extreme condition 1 is that all parents are worst case, and extreme condition 2 is that all are best case. The posterior probabilities of the remaining corresponding child nodes are shown in the screenshot of the table.

For example, when in two extreme conditions, the six states of the fraud types (Consumption fraud, Diction fraud, Identity fraud, Inducement fraud, Other new fraud, Shopping fraud) have probabilities of (0.25, 0.1, 0.3, 0.2, 0.05, 0.1) and (0.1, 0.15, 0.15, 0.3, 0.2, 0.1), respectively. According to the highest index, we can conclude that condition 1 is prone to identification-type fraud and extreme condition 2 is prone to inducement-type fraud. The results of the effects on the number of repeat victimizations, police reports, losses, and victim recovery period of victim can also be obtained from Table 5.

#### 3.4.2. Situational Reasoning

In addition to the two extreme cases considered in the qualitative analysis, a variety of reasonable scenarios should be designed to reason about the electrical fraud results of the parent and independent child nodes. The experimental results states are shown in Table 6. A total of five scenarios were designed for this experiment due to space limitations, and not all possible scenarios were considered.

Scenario 1 is the worst-case scenario with all parent nodes except the fraud-type node. When the fraud-type node changes from shopping-type fraud to identity impersonation-type fraud, the maximum loss probability of the loss scenario node decreases from 6% to 3%.

Scenario 2 is similar to Scenario 1, except that the age type is changed to 70s. The probability of a resultant loss scenario of RMB 300,000 or more rises by 13%.

Scenario 3 is similar to extreme scenario 2, except that the type of scam changes from a lure-type scam to a shopping-type scam. As a result, the probability of losing more than RMB 300,000 decreased from 25% to 6%, and the probability of losing less than RMB 5000 increased from 50% to 52%.

Scenario 4 and Scenario 5 show that the combination of different objective factors changes the scam results accordingly.

#### 3.4.3. Sensitivity Analysis Strategy

Sensitivity analysis is one of the important techniques for testing the probabilistic parameters of causal network BBNs. Specifically, it is achieved by studying the effect of small changes in the numerical parameters of the model (i.e., prior probability and conditional probability) on the resulting parameters (e.g., posterior probability). Therefore, in this paper, based on the calculation of entropy reduction and mutual information, the sensitivity value of each node is calculated by applying the sensitivity analysis in GeNIe software. If the sensitivity value is large, then a small change in that node may lead to a large change in the target posterior probability. If the sensitivity value is small, then the posterior probability hardly changes, even if the node undergoes a large change. The sensitivity values of the leaf node “loss situation” to other nodes are shown in Table 7, where age is the most sensitive category, with a sensitivity value of 13.6%, followed by knowledge level (7.5%), urban economic level (6%), and day and night (5.4%).

According to Table 7, the four most-sensitive factors of the “money loss level” node have been identified as: “age”, “grade”, “city level” and “day and night”. Meanwhile, this paper uses Hu et al. to propose a quantitative form of strategy for reducing potential losses to effectively focus on sensitive risk factors and accurately assess risk safety [45]. The specific process is as follows: (1) First, calculate the posterior probability of the loss scenario node using the input prior probability of the four sensitive factors’ reduction as evidence. (2) Then, the difference between the posterior probability of the risk of the loss scenario above RMB 300,000 and the original posterior probability (13.3%) is determined as the benefit indicator B of the loss risk-reduction strategy. (3) Finally, the cost indicator C is introduced according to Equation (7) [45] and B-C as the assessment indicator to determine the most effective loss risk-reduction strategy.

(7) $$C=\sum_{i=1}^{n}{\left(\frac{p_{i}-p_{i}^{\prime }}{p_{i}}\right)}^{4}$$

where $p_{i}$ is the original prior probability and $p_{i}^{\prime }$ is the reduced prior probability, in which case n is 4.

By comparing the results of strategies 1 to 4 (Table 8), the impact priorities could be identified as knowledge level, diurnal environment, urban economic level, and age. Then, according to the proposed priorities, different reduction allocations in strategies 5 to 8 were designed with a stable total reduction (−0.1). The results show that strategy 6 is the most effective. It reduces the risk of high fraud losses by 0.008 with an evaluation index of 4.9290 × 10^−3^, while it can further be described as the most effective strategy when the total reduction is 0.1, age is 0.01, knowledge level is 0.05, urban economic level is 0.02, and day and night is 0.02.

Several of these points can be learned from the “Network Fraud Trend Analysis Research Report” released by 360’s Security Brain department on 19 March 2020: (1) Men are more likely to be fooled and women suffer greater losses. (2) The victims are younger, with the post-2000s and post-1990s becoming the main victims of fraud. (3) The loss of game fraud shows the characteristics of high, middle, and low end of age, with the greatest loss in the post-1980s. (4) The loss of financial fraud is positively correlated with age, and the older the age, the greater the loss. Since the sensitivity attributes in the above analysis report are approximately the same as the attributes of the sensitivity analysis in this section, the validity of the sensitivity analysis in this paper can be illustrated.

#### 3.4.4. Model Validation

This is because by validating the BBN-based consequence model, a reasonable level of confidence can be provided for the results of the model. Therefore, this study also performs model validation based on the three axioms proposed by Jones et al. to determine whether the model meets the axiomatic requirements, of which the details are as follows [8,46]:

(1) If the prior probability of the parent node decreases/increases slightly, the posterior probability of the corresponding child node should also change accordingly.
(2) As the probability distribution of the parent node changes, the corresponding child nodes should have a consistent impact.
(3) The magnitude of the total impact of the combination of probability changes from m attributes on the value should always be larger than that from the combination of $m-n\left(n\in m\right)$ attributes.

For example, considering the economic level of the parent city in the loss case, when the prior probability of the state “high” is set from 14% to 20%, the probability of maximum loss increases from 7.3% to 7.6%, and the probability of the state “low” in the victim recovery cycle increases from 36.0% to 36.8%. Based on this change, when the prior probability of the parent node’s knowledge level in the “high” state is set from 22% to 42%, the probability of maximum loss increases from 7.6% to 8.0% and the probability of the victim’s recovery cycle state being “low” increases from 36.8% to 37.1%. The change process of each impact node satisfies the triple axiom definition and achieves the partial validation of the model. Similar analysis can be performed for other nodes.

## 4. Example of Model Optimization and Application for a Specific Region

In this section, we will take City S as an example to verify the validity of the model, and modify the node structure of the Bayesian network according to the actual situation of City S to improve the analysis and early warning of telecom fraud in City S.

### 4.1. Data Acquisition

Most of the data related to telecom fraud in City S are open data obtained from open-source sources such as the Internet and government platforms. We imported all data into the database and then deleted the impractical feature data (too many missing values, collection error data) generated by the collection error. The initial database contained 60,000 samples, and 2437 samples were utilized for analysis after screening.

#### 4.1.1. Basic Information of City S

City S has nine municipal districts and one administrative district, with an area of 1997.47 square kilometers, a regional GDP of 323.8768 billion yuan, and a per capita GDP of 183,000 yuan. The education level of 17,681,600 permanent residents in City S in 2021 is unevenly distributed. The number of people with primary and junior high school education accounts for 42.7% of the total number of people, and this group is mostly a foreign population. They have poor living conditions, high commuting costs, often encounter fraud, and have a single way of learning about telecom-fraud prevention information; in addition, about 20.7% of the population has a high school education, 28.8% of the population has a university degree or above, and this group has a better living and working environment, so they are easily exposed to anti-telecommunications fraud propaganda and alerts in their life and work.

#### 4.1.2. Deceived Situation

**Figure 3:**
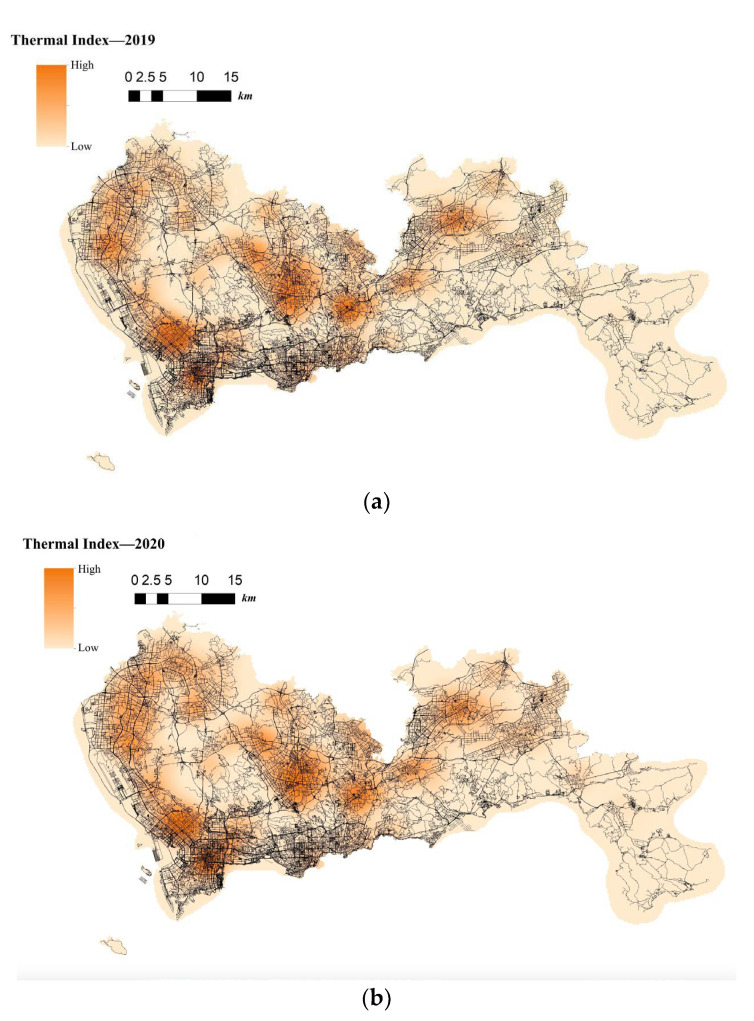

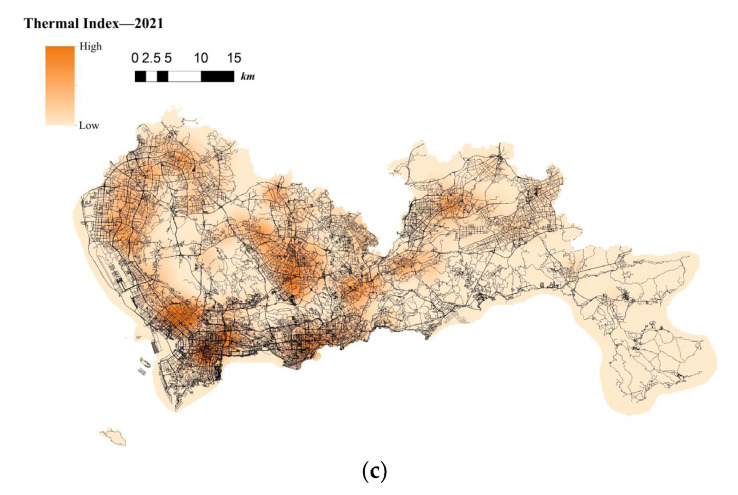
(**a**). Choropleth Map of the Location of Telecom-Fraud Cases in City S in 2019. (**b**). Choropleth Map of the Location of Telecom-Fraud Cases in City S in 2020. (**c**). Choropleth Map of the Location of Telecom-Fraud Cases in City S in 2021.

The situation of telecom fraud in City S is more serious. Relevant statistics show a total of more than 60,000 cases involved in the region for 2019–2021, with an average of more than sixty cases per day. The spatio-temporal visualization of telecom-fraud cases from 2019 to 2021 using ArcGIS software [47] is shown in Figure 3. From the mapped Choropleth Map, it is clear that telecom fraud has the following characteristics: (1) telecom-fraud cases present a road network concomitance in space; for example, in areas with dense road networks, the telecom-fraud cases are intensive; and (2) present dynamic changes in time; for example, in the period of the new COVID-19 virus outbreak, telecom-fraud cases were in high incidence, especially in areas with dense road networks and convenient living.

### 4.2. Bayesian Network Structure Optimization

For the actual situation of City S, this section separately verifies the impact on the telecom-fraud loss situation from various aspects, such as external dynamic factors, major unexpected events, and special time points. The improvement and optimization of the structure of the Bayesian network is realized to make the model structure more suitable for the actual situation of City S.

#### 4.2.1. Bayesian Networks under the Influence of External Dynamic Factors

Taking into account the actual measure taken of telecom fraud in City S, two external dynamic influence factors (police crackdown and propaganda prevention) are added in this section. This section calculates the probability distribution of these two nodes influencing other nodes based on the pre-processed data. The relevant node states are displayed as shown in Table 9.

A type of indirect causation is found in police crackdownsf a significant increase in a certain type of crime is observed in recent months, the police department will generally take a special action to combat it. Such arrests of criminal gangs contribute to a sharp decline in similar crimes in the area. However, due to the cross-regional nature of telecom fraud and the offshore concealment of criminal gangs, there will still be a large number of telecom-fraud cases in the region. Therefore, a dynamic assessment is needed in order to study how changes in police crackdown behavior affect changes in the characteristics of victims.

Experience has taught us that fraud prevention in the community is more effective and spreads better when it is carried out. It therefore contributes to the decrease in the probability of fraud in Bayesian networks, as evidenced by the decrease in the amount of fraud, the increase in the number of police calls, the increase in the vigilance of historical victims, and the decrease in the proportion of repeat victims.

**Figure 4:**
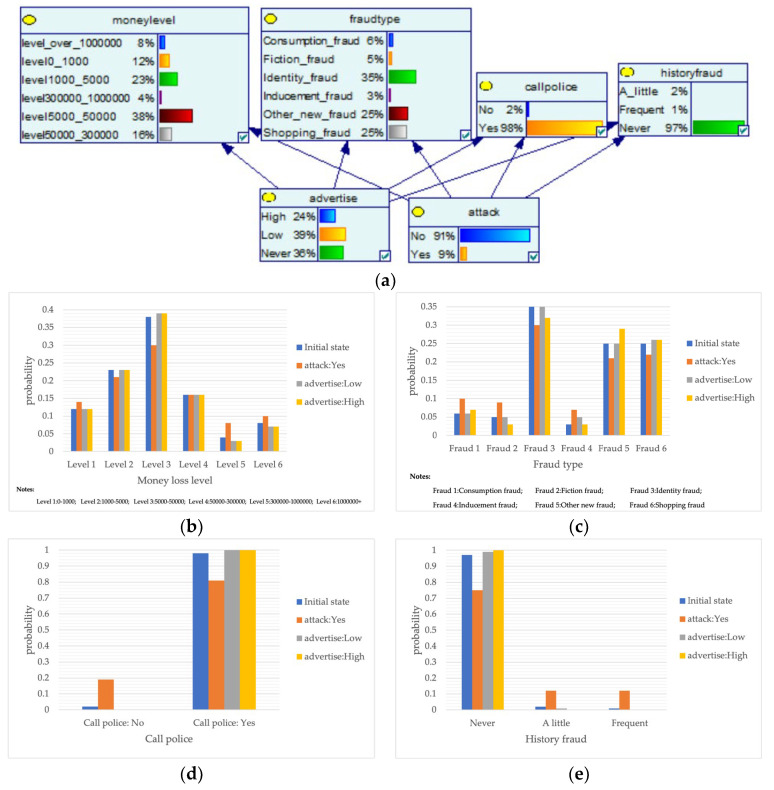
A Bayesian Network Consequence Model for Telecom Fraud Combining External Dynamic Factors. (**a**) Bayesian Network Structure of Telecom Fraud Combining External Dynamic Factors; (**b**) Probability Distribution of “Money Loss Level” Nodes under the Influence of External Dynamic Factors; (**c**) Probability Distribution of “Fraud Type” Nodes under the Influence of External Dynamic Factors; (**d**) Probability Distribution of “Call Police” Nodes under the Influence of External Dynamic Factors; (**e**) Probability Distribution of “History Fraud” Nodes under the Influence of External Dynamic Factors.

Despite the current popularity of dynamic Bayesian networks, this section is built based on static Bayesian networks. Because the impact time of nodes such as police crackdown and anti-fraud propaganda on the consequences of fraud is dynamically fluctuating, the results cannot be effectively measured by time slicing. In order to observe the influence of external dynamic factors, the state of police attack node and anti-fraud advertise node can be changed to observe the probability of a change in loss situation, fraud type, and other related nodes in the Bayesian network model built by combining external dynamic factors. As shown in Figure 4, the anti-fraud advertise variable decreases the probability of loss situation of RMB 300,000 or more from 12% to 10%, and the police attack variable decreases the probability of loss situation of RMB 5000–RMB 50,000 from 38% to 30%, while the high frequency of anti-fraud advertisements reduces the percentage of repeat fraud victims. The correlation results indicate the usefulness of both factors for anti-fraud efforts.

#### 4.2.2. Bayesian Networks for Major Public Health Emergencies

**Figure 5:**
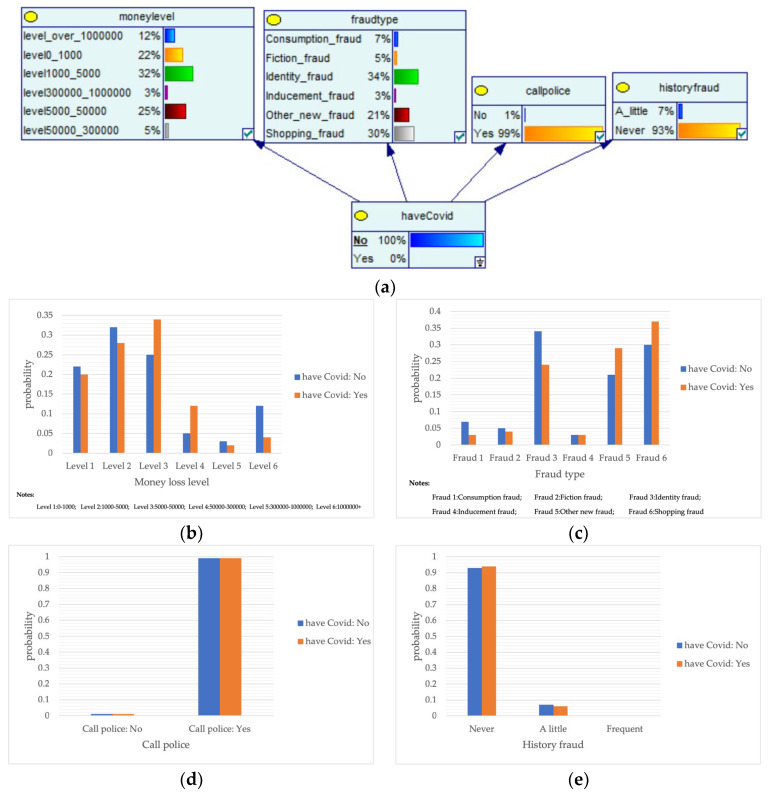
A Bayesian Network Consequence Model of Telecom Fraud Combined with Major Public Health Emergencies. (**a**) Bayesian Network Structure of Telecom Fraud Combining Major Public Health and Safety Emergencies; (**b**) Probability Distribution of “Money Loss Level” Nodes under the Influence of Major Public Health and Safety Emergencies; (**c**) Probability Distribution of “Fraud Type” Nodes under the Influence of Major Public Health and Safety Emergencies; (**d**) Probability Distribution of “Call Police” Nodes under the Influence of Major Public Health and Safety Emergencies; (**e**) Probability Distribution of “History Fraud” Nodes under the Influence of Major Public Health and Safety Emergencies.

The outbreak of COVID-19 and major public health emergencies in 2020 has limited human travel and changed communication methods, while also aggravating the problem of telecom fraud. A Bayesian network model incorporating major public health emergencies was constructed to observe the impact of major public health emergencies on the consequences of fraud, such as types of telecom fraud, losses, and the percentage of repeat victims. The model parameters were learned by selecting data related to the outbreak of the COVID-19 outbreak in City S at the beginning of the outbreak (30 January 2020 to 22 February 2020) and the normal period (30 January 2019 to 22 February 2019) and telecom fraud, and the above impact was observed by reasoning conditional on the evidence. As shown in Figure 5, it can be seen that during the new COVID-19 epidemic, shopping scams rose to 37%, and the probability of incidents of loss between RMB 5000 and 50,000 also rose, from 25% to 34%. The results show that major public health emergencies can have an impact on the form and loss of telecom fraud, so paying attention to the public health emergencies factor can help achieve early perception, timely intervention, and timely combat.

#### 4.2.3. Bayesian Networks for Different Seasons and Time Points

**Figure 6:**
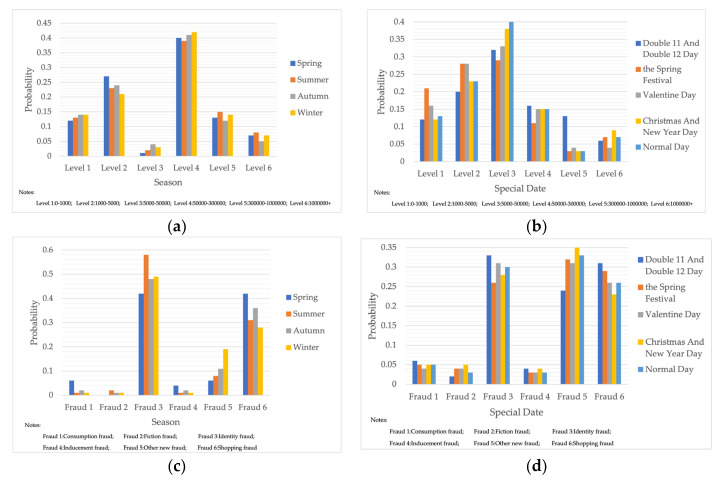
Probability Distribution of Bayesian Network Consequence Model of Telecom Fraud in Different Seasons and Specific Time. (**a**) Seasonal-Loss Node Conditional Probability Distribution Chart; (**b**) Specific Time-Loss Node Conditional Probability Distribution Chart; (**c**) Seasonal-Fraud Type Node Conditional Probability Distribution Chart; (**d**) Specific Time-Fraud Type Node Conditional Probability Distribution Chart.

Telecom fraud also has certain chronological characteristics, and the distribution of telecom-fraud cases’ characteristics is different under different seasons. At the same time, telecom-fraud cases at specific points in time each year are characterized by a surge in specific fraud types, a rise in the number of reported cases, and the victimization of specific groups. Therefore, this section builds out a Bayesian network consequence model of telecom fraud, combining seasons and specific time points. Four seasons (spring, summer, autumn and winter) and seven specific time nodes (Double 11, Double 12, Chinese New Year, Valentine’s Day, Tanabata, Christmas Day, and New Year’s Day) are selected as the states of the seasons and specific time factors, and the impact of the consequences of telecom fraud is observed by changing the node evidence. As shown in Figure 6, under the seasonal factor, telecom fraud shows a distribution pattern of large fraud loss amount in summer and small loss in autumn. The types of fraud show a significant summer season for identity-based frauds and anti-seasonal changes for consumer frauds and fictitious risk-based frauds. Under the specific time node factors, during the Double 11, Double 12 and other online shopping festivals; when the probability of loss situation of more than RMB 300,000 rises; on New Year’s Day; and during the Spring Festival and other reunion periods, fictitious risk class fraud significantly surged. The above changes are consistent with the fact that time and space behaviors produce interactive feedback on telecom fraud. Therefore, adding seasons and specific time nodes can help to improve Bayesian networks effectively from the perspective of time series.

### 4.3. An Early Warning Framework Based on Bayesian Networks and Telecom-Fraud Knowledge Graphs

Based on the actual situation of telecom fraud in City S and combining the analysis results of the above three telecom-fraud Bayesian network consequence models, this section improves the structure of the initial Bayesian network telecom-fraud consequence model in order to achieve a fine-grained risk assessment of telecom fraud and enhance the interpretability of the model. As shown in Table 10, the risk assessment provides granular results on the risk of fraud, including the vulnerability of each potential victim, the predicted level of loss, and the approximate recovery period of the victim. The results can provide a research basis for subsequent telecom-fraud early warning, decision guidance, and targeted intervention by anti-fraud staff.

As well as being informed of the risk probability, the Bayesian network telecom-fraud consequence model can also be technically complemented by combining telecom-fraud knowledge graphs. Both are network structures, where Bayesian networks can easily quantify risk elements but cannot dynamically add knowledge and effectively partition fine-grained nodes. In contrast, mapping can identify the nodal relationships between different elements and answer questions with observational data, but it cannot easily control for multiple confounding factors [48,49]—for example, the relationship between the attributes of different victims, places of victimization, social environment, and the causal relationship between events in different fraud phases. Therefore, a telecom-fraud risk warning system based on probability estimation combined with graphical relationships can increase the accuracy of prediction, realize a hierarchical and graded warning mechanism, and effectively reduce the pushing of content unrelated to risk elements, which helps to provide decision support for police departments and increase the efforts to combat and promote the designated targets.

### 4.4. Limitations

Firstly, the proposed model is not experimentally compared in this paper to verify its validity. Because of the lack of a baseline model and the lack of data collection from un-scammed victims, we use the Bayesian network’s own characteristics and adopt causal inference, sensitivity analysis, and three axioms to verify the validity of the model. Secondly, regarding the geographical problem, this paper only considers the actual situation in City S. However, due to the influence of the economic development gap between different regions, the indicators and probability distribution of the model in different regions need to be further optimized. Finally, there is the cost issue, which is oriented to the large-scale use of telecom-fraud analysis and the early warning framework, as the collection and fusion of data will consume huge amounts of human and material resources. Future research can address the above issues by combining the big data and causal fusion methods proposed by Bareinboim et al., to solve the cost problem due to dynamic adjustments between different geographic areas [50].

## 5. Conclusions

Identifying and assessing the consequences of telecom fraud are important elements of risk management and early warning decisions. This study proposes a BBN consequence model incorporating multivariate heterogeneous data to estimate the impact of victim portrait, crime geography characteristics, social environment portrait, fraud process, and fraud outcome. To effectively estimate the impact of the consequences of telecommunication fraud, a causal model based on Bayesian networks is constructed. The main influencing factors of the model are first identified through a literature review, and then the causal relationships between the parent and child nodes are established based on expert knowledge and the published literature. Scenario analysis can also be used to predict the likely impact and consequence indices for different scenarios, and a sensitivity analysis can be employed to identify influential independent parameters or parent nodes and their impact on the overall consequence index. In order to demonstrate the effectiveness and applicability of the proposed model, this paper also selects City S as the research object for analysis and discussion, using the telecom-fraud risk warning framework.

The BBN-based model of the effects of telecom fraud can provide quantitative and qualitative information about the different influencing factors of telecom fraud. Thus, the framework proposed in this paper can be a unique and valuable tool for the police department to use to prevent telecom fraud. The tool is a type of causal model that can be easily scaled. In addition to the ability to highlight the most sensitive factors in the network, it allows us to flexibly consider more influencing factors, more information, and the priority set by the relevant factors. Additionally, since the effectiveness of the developed model depends on the CPT in the BBN structure, generated based on the expert knowledge, the model can provide the weight or credibility score of the expert to the decision maker for decision support in case of differences in the knowledge and experience of the decision maker. In addition, the source of expert knowledge can be composed of several experts from different disciplines, making the model multidisciplinary. Future-oriented work can also consider the use of dynamic Bayesian network methods to capture changes in outcomes over time for different forms of telecom fraud.

## Figures and Tables

**Table 1:** Causal Hierarchy and Technical Approach Table.

Level	Typical Activity	Typical Questions	Technical Approach
Association	Seeing	What is? How would seeing X change my belief in Y?	BBN ^1^, MC ^2^, SVM ^3^
Intervention	Doing, Intervening	What if? What if I do X?	BBN, MC
Counterfactuals	Imagining, Retrospection	Why? Was it X that caused Y? What if I had acted differently?	BBN

^1^ BBN = Bayesian Belief Networks; ^2^ MC = Markov Chain; ^3^ SVM = Support Vector Machine.

**Table 2:** Bayesian Network Node Design in Telecom-Fraud Risk Model.

Node Classes		Nodes Name (BN Variables)	States of Bayesian Nodes	References
Personalized Portrait Process	Victim Portrait	A. Sex	(1) Female	[8,18,19,20]
			(2) Male	
		B. Era	(1) 00s	
			(2) 90s	
			(3) 80s	
			(4) 70s	
			(5) 60s	
			(6) 50+s	
		C. Grade	(1) Low	[21,22]
			(2) Medium	
			(3) High	
		D. City level	(1) Low	[23,24]
			(2) High	
	Criminal Geography Portrait	E. Region type	(1) Life	[25,26,27]
			(2) Entertainment	
			(3) Work	
			(4) Culture	
			(5) Public services	
			(6) Comprehensive	
		F. Police distance	(1) Near	[28,29]
			(2) Far	
		G. Financial level	(1) Low	[30,31]
			(2) High	
	Social Environment Portrait	H. Day and Night	(1) Night	[32,33]
			(2) Day	
		I. Weather type	(1) Perfect	
			(2) Terrible	
Fraudulent Process		J. Fraud type	(1) Identity fraud	[8]
			(2) Shopping fraud	
			(3) Inducement fraud	
			(4) Fiction fraud	
			(5) Consumption fraud	
			(6) Other new fraud	
		K. History frauded level	(1) Never	[34]
			(2) A little	
			(3) Frequent	
		L. Call police	(1) No	[35]
			(2) Yes	
Fraudulent Results		M. Money loss level	(1) 0–1000	[8,36,37,38]
			(2) 1000–5000	
			(3) 5000–50,000	
			(4) 50,000–300,000	
			(5) 300,000–1,000,000	
			(6) 1,000,000+	
		N. Convalescence period	(1) 0 day	
			(2) 14–30 day	
			(3) 30–180 day	
			(4) more than 180 day	
			(5) more day	

**Table 3:** Regional Function Types—POI Comparison Table.

Region Type	POI Type
Life	residential areas, villas, community centers, dormitories, restaurants and food
Entertainment	shopping consumption, leisure and entertainment, tourist attractions, hotel accommodation
Work	office buildings, industrial buildings, industrial parks, corporate enterprises
Science, Education and Culture	science, education and culture, sports construction
Public Service	health care, living services, transportation facilities, government agencies

**Table 4:** Conditional Probability Table for the Sub-Nodes of “Money Loss Level”.

Nodes						Loss Level						
A ^1^	B ^2^	C ^3^	D ^4^	H ^5^	I ^6^	L ^7^	0 | 1000	1000 | 5000	5000 | 50,000	50,000 | 300,000	300,000 | 1,000,000	1,000,000+
A1	B1	C1	D1	H1	I1	L1	23%	51%	18%	1%	1%	6%
A1	B1	C1	D1	H1	I1	L2	32%	40%	24%	1%	1%	1%
…….						…….						
A2	B1	C1	D1	H1	I1	L1	3%	17%	49%	19%	0%	12%
A2	B1	C1	D1	H1	I1	L2	7%	45%	32%	11%	1%	4%
	……					……						

^1^ A= Sex; ^2^ B = Era; ^3^ C = City level; ^4^ D = Grade; ^5^ H = Day and Night; ^6^ I = Weather type; ^7^ L = Fraud type.

**Table 5:** Extreme Condition Test of Telecom-Fraud Consequence Model Based on BBN.

Nodes Name	Extreme Conditions 1	Extreme Conditions 2
Sex	Female	Male
Era	60s	00s
Grade	Low	High
City level	Low	High
Region type	Life	Culture
Police distance	Far	Near
Financial level	High	Low
Day and Night	Day	Night
Weather type	Terrible	Perfect
Fraud type	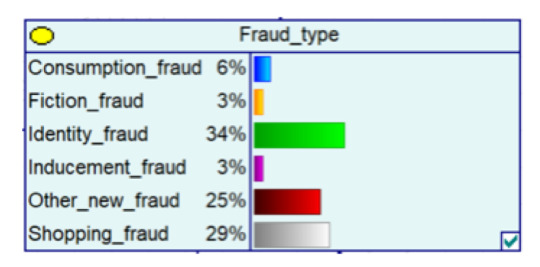	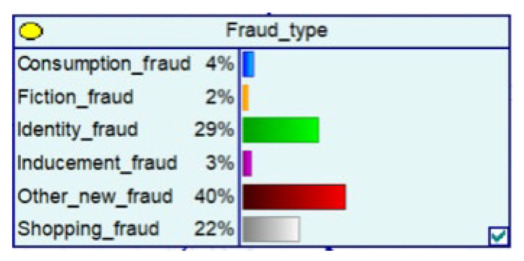
History frauded level	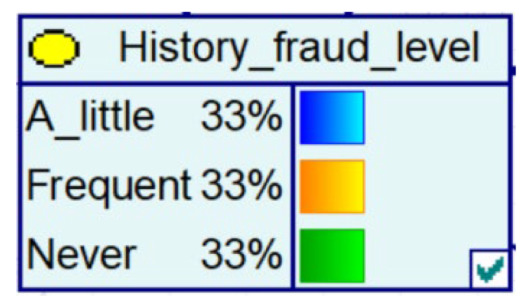	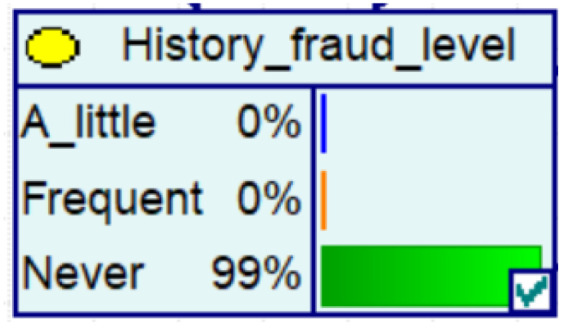
Call police	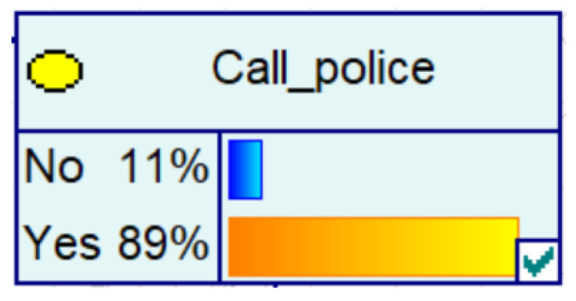	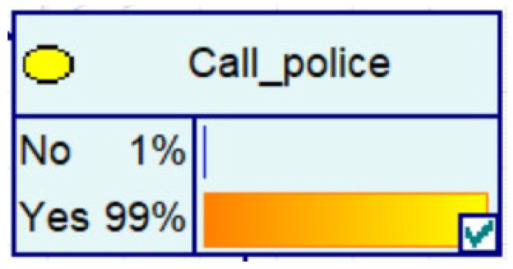
Money loss level	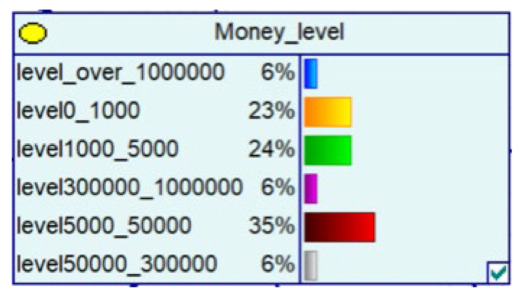	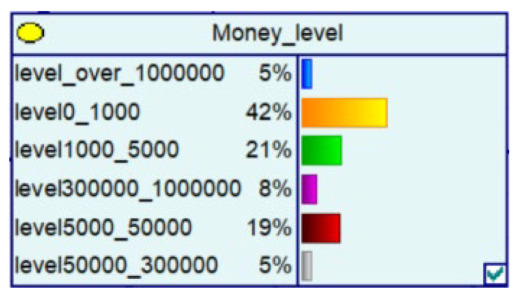
Convalescence period	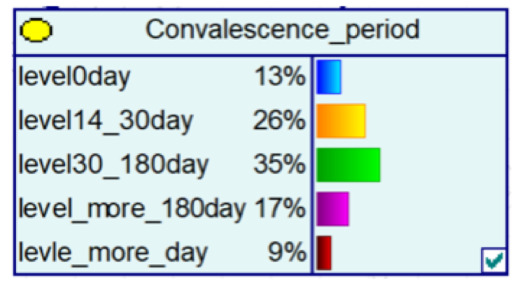	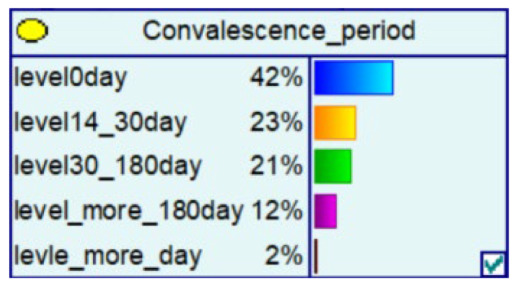

**Table 6:** Scenario Analysis of BBN Based Telecom-Fraud Consequence Model.

Nodes Name	Scenario 1	Scenario 2	Scenario 3	Scenario 4	Scenario 5
Sex	Female	Female	Male	Female	Female
Era	60s	70s	00s	70s	70s
Grade	Low	Low	High	Medium	Low
City level	Low	Low	High	Low	Low
Region type	Life	Life	Culture	Life	Life
Police distance	Far	Far	Near	Far	Far
Financial level	High	High	Low	High	High
Day and Night	Day	Day	Night	Day	Night
Weather type	Terrible	Terrible	Perfect	Terrible	Terrible
Fraud type	Identity fraud	Identity fraud	Shopping fraud	Identity fraud	Identity fraud
Money loss level	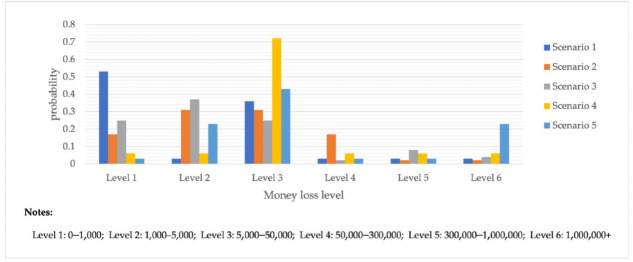				

**Table 7:** Sensitivity Analysis Results of Various Factors to the “Money Loss Level” Variable.

Nodes Name	Sensitivity Values
Sex	1.7%
Era	13.6%
Grade	9.5%
City level	6%
Region type	0.0012%
Police distance	0.00006%
Financial level	0.016%
Day and Night	5.4%
Weather type	5.1%

**Table 8:** Indicator Results under Different Risk-Reduction Strategies.

ID	Sex	Grade	City Level	Day and Night	B	C	B-C
1	−0.05	0	0	0	0.003	0.1090	−0.1060
2	0	−0.05	0	0	0.004	2.4824 × 10^−3^	1.5176 × 10^−3^
3	0	0	−0.05	0	0.004	1.5812 × 10^−2^	−1.1812 × 10^−2^
4	0	0	0	−0.05	0.001	3.5995 × 10^−4^	6.4005 × 10^−4^
5	−0.02	−0.05	−0.01	−0.02	0.008	5.3098 × 10^−3^	2.6902 × 10^−3^
6	−0.01	−0.05	−0.02	−0.02	0.008	3.0710 × 10^−3^	4.9290 × 10^−3^
7	−0.01	−0.06	−0.01	−0.02	0.007	5.3567 × 10^−3^	1.6433 × 10^−3^
8	−0.02	−0.04	−0.01	−0.03	0.007	3.8816 × 10^−3^	3.1184 × 10^−3^

**Table 9:** External Dynamic Factor Node State Design Table.

Node Classes	Nodes Name	States of Nodes
External dynamic factors	Police attack	(1) Yes
		(2) No
	Anti-fraud advertisement	(1) Never
		(2) Low
		(3) High

**Table 10:** Fine-Grained Risk Assessment Table for Potential Victims of Telecom Fraud.

Potential Victims ID	No. 1	No. 2	No. 3	…	No. n
Sex	Male	Female	Male	…	Female
Era	35	61	21		46
Police distance (m)	1498.2	3047.6	1745.1		2245.8
Weather type	Rain	Sun	Rain		Sun
season	Winter	Spring	Summer		Winter
time point	2022.11.11	2022.2.11	2022.8.14		2022.5.14
Region type	Entertainment	Life	Culture		Work
Advertise time	2	0	5		1
Fraud type	Shopping fraud (43%)	Consumption fraud (34%)	Inducement fraud (42%)		Identify fraud (31%)
Money loss level	5000–50,000 (23%)	1000–5000 (24%)	5000–50,000 (38%)		50,000–300,000 (29%)
Convalescence period	14–30 days (33%)	14–30 days (29%)	30–180 days (31%)		30–180 days (24%)

## Data Availability

The data presented in this study are available on request from the corresponding author. The data are not publicly available due to personal privacy.
